# Retraction: One-pot synthesis of Hantzsch dihydropyridines using a highly efficient and stable PdRuNi@GO catalyst

**DOI:** 10.1039/d1ra90151f

**Published:** 2021-10-06

**Authors:** Laura Fisher

**Affiliations:** Royal Society of Chemistry Thomas Graham House, Science Park, Milton Road Cambridge UK advances-rsc@rsc.org

## Abstract

Retraction of ‘One-pot synthesis of Hantzsch dihydropyridines using a highly efficient and stable PdRuNi@GO catalyst’ by Tuna Demirci *et al.*, *RSC Adv.*, 2016, **6**, 76948–76956. DOI: 10.1039/C6RA13142E.

The Royal Society of Chemistry hereby wholly retracts this *RSC Advances* article due to concerns with the reliability of the data in the published article.

The particle size histogram of PdRuNi@GO NPs in Fig. 2c has the same relative bar heights as particle size histograms in at least six other papers by the same authors, all representing different nanoparticles or synthetic methods. The authors provided replacement data for consideration. However, an expert reviewed the authors’ response and concluded that it did not satisfactorily address the concerns, and that the replacement figure did not fully support the conclusions.

The 2D Pd3d XPS spectra for PdRuNi@GO nanoparticles in Fig. 3a was later published by the authors in *Scientific Reports* in Fig. 2d to represent PdNi@rGO.^1^ The 3D Pd3d XPS spectra for PdRuNi@GO nanoparticles in Fig. 3a was later published by the authors in *Nano-Structures & Nano-Objects* in Fig. 2a to represent PdRu@GO nanocomposites.^2^

The 3D Ru3p XPS spectra for PdRuNi@GO nanoparticles in Fig. 3b was later published by the authors in *Nano-Structures & Nano-Objects* in Fig. 2b to represent PdRu@GO nanocomposites,^2^ in the *Journal of Molecular Liquids* in Fig. 3b to represent Ru(0)/graphite NPs,^3^ in *Nanocarbon and its Composites* in Fig. 3b to represent Ru/PVP@C^4^ and previously by the authors in *Chemistry Select* in Fig. 3b to represent RuPd@GO NPs.^5^

The 3D Ni2p XPS spectra for PdRuNi@GO nanoparticles in Fig. 3b was later published by the authors in *Biosensors & Bioelectronics* in Fig. 2b to represent Ni@f-MWCNT.^6^ The authors claim that the XPS spectra in Fig. 3 were included in error and provided replacement data for consideration. However, an expert reviewed the author’s response and concluded that it did not satisfactorily address the concerns, and that the replacement figure did not fully support the conclusions.

Given the significance of the concerns about the validity of the data, the findings presented in this paper are no longer reliable.

Sinan Eriş, Tuna Demirci, Mustafa Arslan, Benan Kilbas and Fatih Sen oppose this retraction. Betül Çelik and Yunus Yıldız were contacted but did not respond.

Signed: Laura Fisher, Executive Editor, *RSC Advances*

Date: 23^rd^ September 2021

## Supplementary Material
